# Association of Biomarker Discrepancy and Treatment Decision, Disease Outcome in Recurrent/Metastatic Breast Cancer Patients

**DOI:** 10.3389/fonc.2021.638619

**Published:** 2021-07-01

**Authors:** Yujie Lu, Yiwei Tong, Xiaosong Chen, Kunwei Shen

**Affiliations:** Department of General Surgery, Comprehensive Breast Health Center, Ruijin Hospital, Shanghai Jiao Tong University School of Medicine, Shanghai, China

**Keywords:** breast cancer, biomarkers, treatment, recurrent, prognosis, discrepancy

## Abstract

**Background:**

Biomarker discrepancy between primary and recurrent/metastatic breast cancer is well known, however its impact on prognosis and treatment after relapse is still unclear. Current study aims to evaluate biomarkers discrepancy between primary and recurrent/metastatic lesions as well as to investigate its association with following treatment pattern and disease outcome.

**Patients and methods:**

We retrospectively included consecutive breast cancer patients undergoing surgery in our center from Jan. 2009 to Dec. 2016 and reported disease recurrence. Patients with re-biopsy and paired biomarkers statuses on primary and recurrent/metastatic lesions were further analyzed. Kappa test was used to analyze the concordance rate of estrogen receptor (ER), progesterone receptor (PR) and human epidermal growth factor receptor-2 (HER2) status. Post-recurrence survival (PRS) was compared between subgroups by Kaplan-Meier curve. Cox regression model was applied to identify impact factors for PRS.

**Results:**

A total of 156 patients were finally included, of whom 70 and 86 had loco-regional and distant recurrence, respectively. Concordance rates of ER, PR and HER2 were 83.3%, 66.7%, and 97.1%, respectively, which was similarly distributed among different recurrent sites (all *P >* 0.05). Primary ER-positivity (*vs* ER-negativity, *P* = 0.014) and loco-regional recurrence (*vs* distant metastasis, *P* = 0.001) were independently associated with superior PRS, while patients with visceral metastasis (*P* < 0.001) had the worst disease outcome. Hormone receptor/HER2 status discrepancy was observed in 28 patients. Fifteen of them changed systemic treatment based on biomarker statuses of recurrent lesion, however, their PRS was not improved compared to those 13 patients who continued the same treatment according to primary biomarkers statuses (*P* = 0.298).

**Conclusion:**

Biomarker discrepancy was observed between primary and recurrent/metastatic breast cancer lesions and had certain influence on treatment strategies after relapse. However, its impact on disease outcome wasn’t established in the current study, which deserves further evaluation.

## Introduction

Breast cancer is the second most common cancer all over the world and the most common malignancy in women. An estimated 2.09 million women are newly diagnosed annually (1). As a heterogeneous disease, breast cancer can be classified into different molecular subtypes according to estrogen receptor (ER), progesterone receptor (PR), and human epidermal growth factor receptor-2 (HER2) status, which resulted in individualized treatment (2). However, 20-30% early breast cancer patients will relapse despite optimate comprehensive treatment (3, 4), which is considered a major cause of breast cancer related death (5).

Systemic treatment of recurrent/metastatic breast cancer is traditionally based on primary tumor biomarker statuses, with a five-year overall survival (OS) rate at 27% (6). However, numerous studies have demonstrated that substantial discrepancy of ER, PR, and HER2 status exists between primary and recurrent/metastatic tumors, which may influence disease outcome. Schrijver et al. reported the discordance rates of ER, PR, and HER2 were 19.3%, 30.9%, and 10.3%, respectively, in a meta-analysis of 39 studies (7). Dieci et al. and Shiino et al. had both demonstrated that loss of receptors, which is defined as positive in primary tumor and negative in recurrent lesion, leads to a worse survival (8–10). On the contrary, however, Amir et al. found that hormone receptor (HoR) and HER2 status discrepancy is not associated with patients’ disease outcome in a prospective analysis (11). The discrepancy rates of biomarkers were variable and its impact on survival was still lack of strong evidence. In regard to its influence on treatment, some other studies indicated that in 14-18% cases, subsequent systemic strategy may be changed according to biomarker statuses of recurrent/metastatic tumor (8, 11, 12). However, few of these studies put emphasis on the association between biomarker discrepancy and clinical outcome after recurrence. In fact, in the meta-analysis of Schrijver et al., there were 14-62% and 67% patients changed their treatment corresponding to HoR and HER2 discrepancy between primary and recurrent/metastatic tumors (7). So, further analysis is needed to evaluate biomarker discrepancy between primary and recurrent/metastatic breast cancer as well as to investigate its association with subsequent treatment pattern and disease outcome.

According to the aforementioned evidence, nowadays, it is recommended by several clinical guidelines that first recurrence disease should be re-biopsied to confirm pathology diagnosis and re-assess ER, PR and HER2 status on recurrent/metastatic tumor if possible (13–16). Meanwhile, there is no consensus whether re-biopsy of recurrent/metastatic lesions should guide subsequent treatment decisions and it is still unclear if biomarker discrepancy has any influence on further disease outcome.

In current study, we aim to evaluate the concordance rates of ER, PR and HER2 statuses between primary and recurrent/metastatic breast cancer lesions, to investigate its association with following systemic treatment and post-recurrence survival (PRS) in recurrent/metastatic breast cancer patients.

## Patients and Methods

### Study Population

Continuous patients undergoing surgery in the Comprehensive Breast Health Center, Ruijin Hospital, Shanghai Jiao Tong University School of Medicine, Shanghai, China from Jan. 2009 to Dec. 2016 were retrospectively included. Inclusion criteria were as follows: 1) histologically diagnosed breast cancer patients, 2) occurrence of loco-regional recurrence (LRR) or distant metastasis during follow-up, 3) histo-pathological analysis of recurrent/metastatic lesions by biopsy or resection 4) complete follow-up. *De novo* Stage IV patients were excluded. All clinical information was obtained from Shanghai Jiao Tong University Breast Cancer Database (SJTU-BCDB). This approach was approved by the independent Ethical Committees of Ruijin Hospital, Shanghai Jiao Tong University School of Medicine, and was in accordance with the Helsinki Declaration. Patient consent to review their medical records was waived by the Ethical Committee of Ruijin Hospital in case of retrospective study. Meantime, patients included were anonymous, and all medical data of patients were kept confidential.

### Tumor Histo-Pathologic Evaluation

Histo-pathologic evaluation of both primary and recurrent/metastatic tumor was accomplished by at least two independent pathologists in the Department of Pathology, Ruijin Hospital, Shanghai Jiao Tong University School of Medicine, Shanghai, China. For patients receiving neo-adjuvant therapy (NAT), post-NAT surgical sample was used for histo-pathologic and immunohistochemical (IHC) assessment. The biomarker statuses taken into analysis are based on the criteria and the initial interpretation at the time of disease diagnosis. Positivity criteria adopted for IHC assessment of ER, PR, and Ki67 were described in our previous report (17). The 2013 ASCO/CAP HER2 testing guideline was adopted to classify HER2 status. Patients with HER2 IHC 2+, fluorescence *in situ* hybridization (FISH) equivocal (HER2/CEP17 ratio < 2.0 with average HER2 gene copy number 4.0-5.9 signals/cell) or no available FISH result were classified as “HER2 uncertain”.

### Treatment in Adjuvant and Recurrent Setting

All enrolled patients underwent standard surgical procedure for their primary tumor in our center with or without neo-adjuvant therapy. Adjuvant treatment strategy was decided through a multidisciplinary team (MDT) meeting with the attendance of surgical oncologists, medical oncologists, radiation oncologists, and breast cancer specialized nurses. Upon suspicious disease recurrence, patients would be recommended to receive radiology-guided biopsy or resection. Another multidisciplinary team meeting would be held to decide the subsequent systemic treatment after disease relapse, based on both primary and recurrent/metastatic disease features.

### Follow-up

Patient follow-up was carried out by specialized nurses. OS was defined as the period between the date of operation and death of any cause or the last follow-up. Disease-free interval (DFI) was computed till the first proven event including LRR and distant metastasis in any sites. PRS was calculated from the date of first recurrence till death of any cause or the last follow-up. The latest follow-up was conducted in May 2019.

### Statistical Analysis

Chi-squared test or Fisher’s exact test were used to descript baseline characteristics of categorical variables among the whole cohort. Concordance rates of ER, PR, and HER2 between primary and recurrent/metastatic lesions were tested by using Kappa test. A Kappa value ≥ 0.6 was considered as a strong concordance, while ≤ 0.4 as a weak concordance (18). Chi-square test and multivariate logistic regression were used to describe baseline characteristics of the study population and to identify impact factors for receptor conversion. PRS were compared between subgroups by Kaplan-Meier curve. Cox regression model was applied to identify impact factors for PRS. All statistical tests were accomplished by IBM SPSS statistics software version 22.0 (SPSS, Inc., Chicago, IL). Figures were produced with GraphPad Prism version 7.0 (GraphPad Software, CA, USA). Two-side P value < 0.05 was considered statistically significant.

## Results

### Patient Demographics

Overall, 5856 continuous patients were diagnosed and underwent breast cancer surgery from January 2009 to December 2016 in our center. A total of 482 patients reported recurrent/metastatic event(s) during follow-up, and 218 of them underwent re-biopsy. Patients receiving fine-needle aspiration biopsy were excluded due to unavailable IHC results. Finally, 156 patients with paired IHC results of ER, PR and HER2 on both primary and recurrent/metastatic lesions were included in analysis (**Supplementary Figure S1**). Baseline patient characteristics were presented in **Table 1**. Mean age at diagnosis was 52.2 years (range 24.0 – 82.0; **Table 1**). The majority of enrolled patients were diagnosed as invasive ductal carcinoma (IDC), 20 ductal carcinoma in situ, 5 invasive lobular carcinoma and other 4 were diagnosed as special type breast cancer including sarcoma, apocrine carcinoma and mucinous adenocarcinoma. Neoadjuvant treatment (NAT) was conducted in thirty patients, 27 of them received neoadjuvant chemotherapy (CT, **Supplementary Table S1**), and none of them reached pathological complete response. Forty-two patients underwent breast-conserving surgery (BCS) and axillary lymph node dissection (ALND) was conducted in 105 patients. Lymph node involvement was found in 75 patients. Almost half patients had grade 3 tumors. Seventy patients had LRR, of whom 28 ipsilateral breast tumor recurrence, 25 chest wall recurrence and 18 regional node recurrence. Besides, 47 and 39 patients had metastases in viscera and bone or soft tissues, respectively.

**Table 1:** Baseline clinico-pathological characteristics of breast cancer patients.

Characteristics	N	%
**Median age, years (range)**	52.0 (24-82)	
**Age, years**		
<50	67	42.9
≥50	89	57.1
**Menstrual status**		
Pre/peri-menopausal	72	46.2
Post-menopausal	84	53.8
**Neoadjuvant treatment**		
Yes	30	19.2
No	126	80.8
**Breast surgery**		
Mastectomy	114	73.1
BCS	42	26.9
**Axillary surgery**		
None	6	3.8
SLNB	45	28.8
ALND	105	67.3
**Histological type**		
IDC	127	81.4
Non-IDC	29	18.6
**Histological grade**		
I-II	72	46.2
III	77	49.4
NA	7	4.5
**pT**		
is	20	12.8
1-2	126	80.8
3-4	10	6.4
**pN**		
0, x*	81	51.9
1-3	75	48.1
**ER status**		
Positive	94	60.3
Negative	62	39.7
**PR status**		
Positive	58	37.2
Negative	98	62.8
**HER2 status**		
Positive	42	26.9
Negative	98	62.8
Uncertain**	16	10.3
**Ki67 status**		
<14%	50	32.1
≥14%	106	67.9
**DFI**		
<2 years	59	37.8
≥2 years	97	62.2
**Recurrent Site**		
LRR	70	44.9
Viscera	52	33.3
Bone or soft tissue	34	21.8

*pN was not available in 6 patients who did not have axillary surgery.

**16 patients were defined as HER2 2+ in IHC test but did not undergo FISH testing.

BCS, breast-conserving surgery; SLNB, sentinel lymph node biopsy; ALND, axillary lymph node dissection; IDC, invasive ductal carcinoma; NA, not available; pT, pathological tumor size stage; pN, pathological lymph node stage; is, in situs; ER, estrogen receptor; PR, progesterone receptor; HER2, human epidermal growth factor receptor-2; DFI, disease free interval; LRR, loco-regional recurrence.

### Concordance of ER, PR, and HER2 Status

All 156 patients had detailed ER and PR statuses in both primary and recurrent/metastatic lesions. However, HER2 and Ki67 discrepancy could not be analyzed in 52 and 11 patients. In detail, 6 and 31 patients were “HER2 uncertain” in primary or recurrent tumor. And in another 5 and 11 patients, HER2 or Ki67 status was not assessable due to restricted quality of re-biopsy sample.

**Figure 1:**
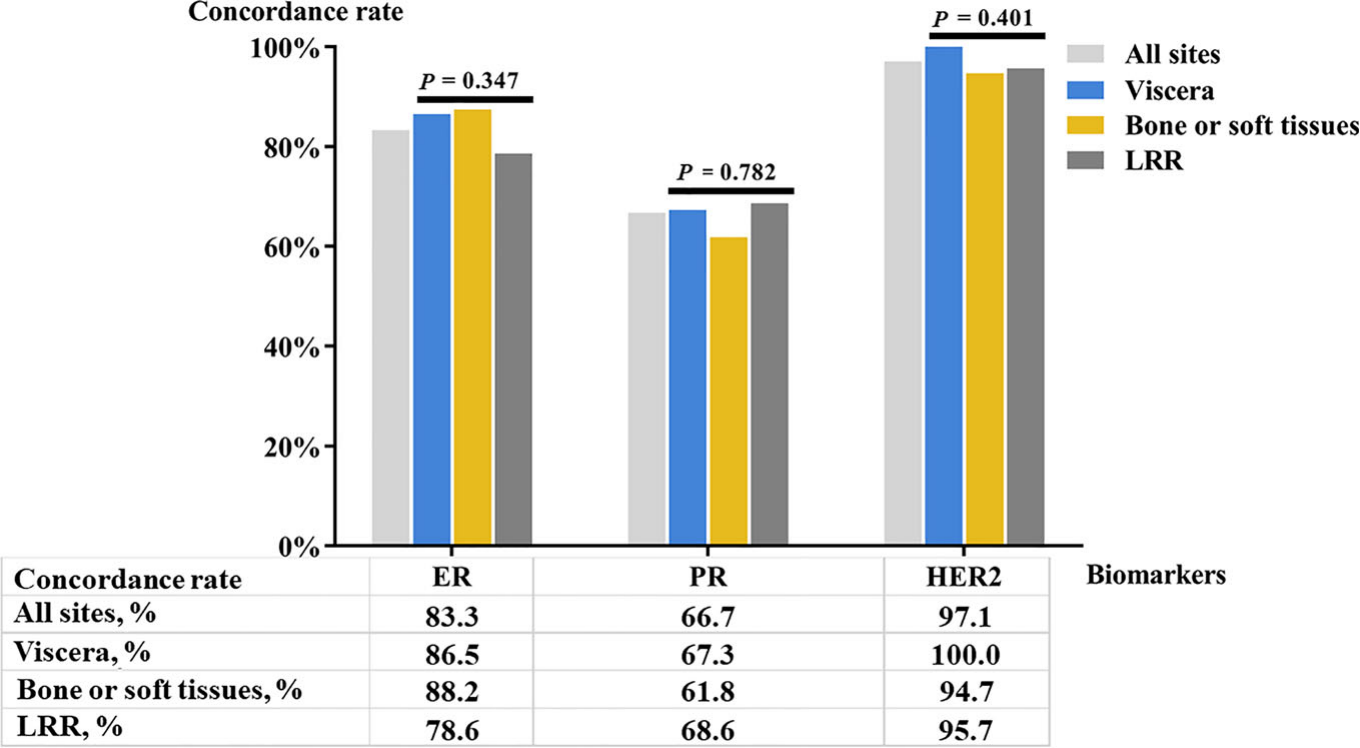
Concordance rate of molecular biomarker status in different recurrent sites. LRR, loco-regional recurrence.

Positivity rates of ER and PR in the primary lesion were 60.3% and 37.2% (**Table 1**). Thirty-five patients had primary HER2-positive disease, while 16 patients were HER2 uncertain in primary lesions. In the recurrent/metastatic lesions, ER and PR positivity was seen in 60.3% and 34.6% patients, and 36.5% patients were HER2-positive. Concordance rates of ER, PR, and HER2 status were 83.3% (κ = 0652, *P* < 0.001; **Table 2**), 66.7% (κ = 0.276, *P =* 0.001), and 97.1% (κ = 0.937, *P* < 0.001), respectively. Proportion of patients with Ki67 ≥ 14% was 69.7% and 75.2% in primary and recurrent/metastatic lesions, respectively, with a concordance rate of 68.3% (κ = 0.152, *P =* 0.169). Concordance rates of ER in LRR, bone or soft tissues, and visceral metastatic lesions were 78.6%, 88.2%, and 86.5%, respectively. PR conversion was observed in 38.2% patients with bone or soft tissues metastasis, while fewer PR conversion was reported in LRR (31.4%) or visceral metastatic (32.7%) patients. No visceral metastasis patients experienced HER2 conversion and the concordance rate of HER2 was 95.7% in LRR patients. There was no significant difference in concordance rates of ER (*P =* 0.347), PR (*P =* 0.782), and HER2 (*P =* 0.401) among different recurrent sites (**Figure 1**).

**Table 2:** Concordance rate of biomarkers between primary and recurrent/metastatic breast cancer lesions.

Primary lesion	N	Recurrent lesion		Concordance rate	Kappa	*P* value
		Positive*	Negative*			
**ER status**	156			83.3%	0.652	**<0.001**
Positive		81	13			
Negative		13	49			
**PR status**	156			66.7%	0.276	**0.001**
Positive		30	28			
Negative		24	74			
**HER2 status**	104^**^			97.1%	0.937	**<0.001**
Positive		35	0			
Negative		3	66			
**Ki67 status**	145^**^			68.3%	0.152	0.169
≥14%		87	14			
<14%		32	12			

*Positive group meant ≥14% in Ki67 status, and negative group meant <14% in Ki67 status.

ER, estrogen receptor; PR, progesterone receptor; HER2, human epidermal growth factor receptor-2.

**HER2 and Ki67 discrepancy were unanalyzable in 52 and 11 patients due to “HER2 certain” or restricted quality of re-biopsy sample.

The bold values mean the difference is statistically significant.

### Factors Associated With Biomarker Discrepancy

Univariate analysis found that age (*P =* 0.036; **Supplementary Table S2**), menstrual status (*P =* 0.031), primary axillary surgery (*P =* 0.027), and pathological lymph node status (*P =* 0.018) were associated with ER conversion between primary and recurrent/metastatic lesions. Histological grade was associated with PR conversion (*P =* 0.030) and no clinico-pathological factor was related with HER2 conversion (all *P >* 0.05).

With regards to adjuvant therapy application, we found that significantly more patients receiving adjuvant endocrine therapy (ET, *P* = 0.012, **Supplementary Table S3**) experienced PR conversion at disease relapse. ER and HER2 conversion were not influenced by adjuvant therapy (all *P* > 0.05).

Further multivariate analysis showed that only node-negative tumor was statistically more likely to experience ER conversion after recurrence (9.3% *vs* 23.5%, odds ratio [OR] = 0.36, 95% confidence interval [CI] = 0.14 - 0.93, *P* = 0.035; **Supplementary Table S4**). What’s more, adjuvant ET application was proven an independent factor of PR conversion, patients receiving adjuvant ET were more likely to have PR discrepancy after relapse (OR = 2.45, 95%CI = 1.17 - 5.12, *P* = 0.017, **Supplementary Table S5**).

### Biomarker Discrepancy and Factors Associated With Survival in Recurrent/Metastatic Breast Cancer Patients

At a median follow-up time of 52.8 months (range 12.5 - 110.6) and a median post-recurrence follow-up time of 20.4 months (range 2.40 - 78.13 months), the median DFI was 31.4 months (range 2.43 - 106.87). Thirty-six patients died after disease relapse. Five-year OS and PRS rates were 77.6% and 52.3%.

**Figure 2:**
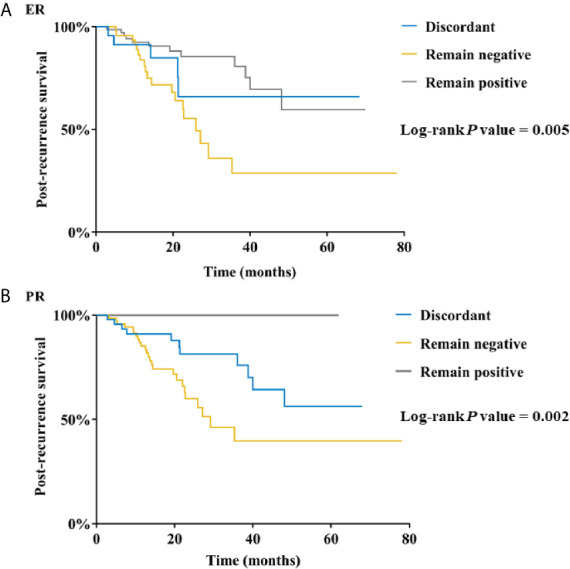
Post-recurrence survival in breast cancer patients according to **(A)** ER conversion and **(B)** PR conversion. ER, estrogen receptor; PR, progesterone receptor.

Univariate analysis demonstrated that breast surgery (*P =* 0.005; **Supplementary Table S6**), axillary node involvement (*P =* 0.013), tumor size (*P =* 0.019), and primary ER status (*P =* 0.007) were associated with PRS. Other impact factors of PRS including recurrent site (*P =* 0.014), ER (*P =* 0.005; **Figure 2**) and PR (*P =* 0.002) conversion between primary and recurrent/metastatic lesions, as well as DFI (*P =* 0.042). No significant different influence on survival was observed between ER-gain (from ER-negative to positive) and ER-loss (from ER-positive to negative) patients (**Supplementary Figure S2**). Further multivariate analysis demonstrated that primary ER status (*P =* 0.014; **Table 3**) and recurrent site (*P =* 0.001) were independently associated with PRS. Patients with visceral metastasis (hazard ratio [HR] = 6.69, 95%CI = 2.50 - 17.87, *P* < 0.001) or bone or soft tissues metastasis (HR = 4.52, 95%CI = 1.57 - 13.04, *P =* 0.005) had a worse PRS compared to LRR patients. Worse PRS was also observed in primary ER-negative tumors compared to ER-positive ones (HR = 2.30, 95%CI = 1.18 - 4.48, *P =* 0.014).

**Table 3:** Multivariate analysis of factors associated with post-recurrence survival in breast cancer patients.

Clinico-pathologic Characteristics	HR	95%CI	*P* value
**Breast surgery**			0.289
Mastectomy	1.00		
BCS	0.39	0.07 - 2.23	
**Axillary surgery**			0.625
ALND	1.00		
SLNB	0.51	0.19 - 2.01	0.333
None	0.00	0 - +∞	0.978
**pT**			0.195
3-4	1.00		
1-2	0.38	0.12 - 1.17	0.091
is	0.26	0.04 - 1.58	0.142
**ER status**			**0.014**
Positive	1.00		
Negative	2.30	1.18 - 4.48	
**Recurrent site**			**0.001**
LRR	1.00		
Viscera	6.69	2.50 - 17.87	**<0.001**
Bone or soft tissues	4.52	1.57 - 13.04	**0.005**
**ER conversion**			0.375
Remain negative	1.00		
Discordant	1.00	0.11 - 9.47	1.000
Remain positive	0.41	0.03 - 5.27	0.496
**PR conversion**			0.934
Remain negative	1.00		
Discordant	0.82	0.29 - 2.36	0.714
Remain positive	0.00	0 - +∞	0.956
**DFI**			0.792
<2years	1.00		
≥2years	1.12	0.49 - 2.57	

OR, odds ratio; CI, confidence interval; BCS, breast-conserving surgery; ALND, axillary lymph node dissection; SLNB, sentinel lymph node biopsy; is, in situs; ER, estrogen receptor; LRR, loco-regional recurrence; pT, pathological tumor size stage; is, in situs; PR, progesterone receptor; DFI, disease-free interval.

The bold values mean the difference is statistically significant.

### Subsequent Treatment Decision and Survival of Patients With Biomarker Discrepancy

**Figure 3:**
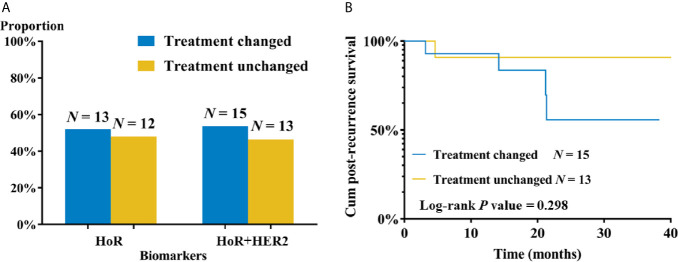
Subsequent treatment and clinical outcome of recurrent’/metastatic breast cancer patients with molecular biomarker discrepancy. **(A)** Subsequent treatment changes according to molecular biomarker conversion. **(B)** Post-recurrence survival by subsequent treatment change. HoR, hormone receptor; HER2, human epidermal growth factor receptor-2.

A total of 28 patients had receptor conversion between primary and recurrent/metastatic lesions (**Figure 3A**), and their detailed systemic treatment information in both adjuvant and post-recurrence setting was listed in the **Table 4**. HoR conversion was observed in 25 patients, including 12 patients from HoR-positive to negative, and other 13 patients vice versa. Three patients had HER2 conversion, all from HER2-negative to positive. Thirteen patients (52.0%) changed their subsequent ET according to new HoR status of recurrent lesions and two patients added HER2-targeted treatment after relapse. Among 12 HoR-loss (from HoR-positive to negative) patients, 8 of them (66.7%) changed following endocrine treatment, while only 5 in 13 HoR-gain (from HoR-negative to positive) patients (38.5%) did so (**Supplementary Table S7**). The 2-year PRS rates of treatment-changed and treatment-unchanged patients were 48.1% and 90.0%. Moreover, the Kaplan-Meier curve exhibited no significant difference in PRS between two groups of patients (*P =* 0.298; **Figure 3B**).

**Table 4:** Detailed systemic treatment information before and after recurrence of patient with biomarker discrepancy.

ID	HoR conversion	HER2 conversion	Recurrent event	Treatment before recurrence	Treatment after recurrence
42	+/-	-/-	Liver	Letrozole	Fulvestrant
420	+/-	+/+	RNR	Letrozole	No ET
454	+/-	+/+	IBTR	Tamoxifen	No ET
513	-/+	NA	RNR	No ET	No ET
781	-/+	NA	IBTR	No ET	No ET
959	+/-	-/-	RNR	Tamoxifen+Goserelin	No ET
989	-/+	-/-	RNR	No ET	No ET
1146	+/+	-/+	Lymph node	No anti-HER2	No anti-HER2
1166	-/+	NA	CWR	No ET	No ET
1226	-/+	+/+	IBTR	No ET	No ET
1915	-/+	NA	RNR	No ET	No ET
2597	-/+	-/-	IBTR	No ET	Tamoxifen+Goserelin
3292	-/+	-/-	CWR	No ET	Letrozole
3652	-/+	NA	IBTR	No ET	Toremifene
3660	+/-	-/-	Lung	Toremifene	Fulvestrant
4209	-/+	NA	CWR	No ET	No ET
4219	+/-	NA	IBTR	Tamoxifen	No ET
4327	+/-	NA	Bone or soft tissue	Tamoxifen	Fulvestrant
4362	+/-	-/-	Bone or soft tissue	Letrozole	No ET
4428	-/+	NA	Bone or soft tissue	No ET	No ET
4603	+/+	-/+	RNR	No anti-HER2	Trastuzmab
4764	+/-	NA	Liver	Letrozole	No ET
5276	+/-	+/+	IBTR	Tamoxifen	Toremifene
6169	+/-	-/-	Lung	Toremifene	No ET
6418	-/+	NA	Liver	No ET	Letrozole
7500	+/+	-/+	CWR	No anti-HER2	Trastuzmab+Pertuzumab
7547	+/-	+/+	Lung	Letrozole	No ET
7978	-/+	-/-	Liver	No ET	Tamoxifen

HoR, hormone receptor; HER2, human epidermal growth factor receptor-2; +/-, from positive to negative; -/-, remain negative; +/+, remain positive; RNR, regional node recurrence; ET, endocrine therapy; IBTR, ipsilateral breast tumor recurrence; -/+, from negative to positive; NA, not available; CWR, chest wall recurrence.

## Discussion

In the current study, we included 156 patients and found that concordance rates of ER, PR, and, HER2 status were 83.3%, 66.7%, and 97.1% between primary and recurrent/metastatic tumors, respectively. After disease relapse, primary ER-negative tumor and distant metastasis were independently associated with worse PRS. Twenty-eight patients (17.9%) had biomarker discrepancy between primary and recurrent/metastatic tumor, and 15 patients changed subsequent treatment according to new receptor status, whose PRS was not superior to those maintaining treatment strategy according to biomarker statuses of primary lesion.

An earlier study of our center declared considerable rates of ER and PR discordance between primary and recurrent/metastatic breast cancer as 14.6% and 16.7%, and only 8.3% patients showed HER2 discrepancy in status in a small cohort of 48 patients (19). As shown in a meta-analysis summarizing 47 articles from 1983 to 2011, the pooled discordance rates were 20%, 33%, and 8% for ER, PR, and HER2 status between primary and metastatic lesions (20). Yeung et al. showed similar findings based on data from 47 studies that median conversion rates of ER, PR, and HER2 at 14%, 21%, and 10%, respectively (21). Moreover, among these receptor statuses, the lowest concordance rate was observed in PR (8, 11, 22, 23) and HER2 status is the most stable one between primary and recurrent/metastatic lesions (24). For patients with HER2 discrepancy, more patients were “HER2 gain” (from HER2-negative to positive) rather than “HER2-loss” (from HER2-positive to negative), according to another meta-analysis (25). McAnena et al. demonstrated in a retrospective study of 132 recurrent breast cancer patients that biomarker discrepancy was observed more in visceral metastasis than bone or soft tissues metastasis (22.0% *vs* 15.2%) (26). However, a more recent research declared that conversion rates of ER, PR, and HER2 were not statistically significantly different among patients with different recurrent sites and times of recurrence (27). In our current study, we included 156 patients with a longer follow-up time to get more convictive result. Single receptor discordant rates were 16.7%, 33.3%, and 2.9% for ER, PR, and HER2, respectively, which was similar to previous studies. All the patients with HER2 discrepancy were from HER2-negative to HER2-positive. Besides, we did not identify significant difference in recurrence site-specific receptor discordant rate, which was also in consistent with a previous study (28).

The mechanism of biomarker discrepancy between primary and recurrent/metastatic breast cancer is still uncertain. Several hypotheses have been proposed, including selective effect of previous treatments (29, 30), intra-tumoral heterogeneity (31), switch in tumor biology (32, 33), and clonal genome evolution (20, 34, 35). Besides, the lack of reproducibility of IHC assays due to pre-analytical and analytical errors is also recognized as a potential explanation for biomarker discrepancy (36, 37). The biomarker discrepancy between primary and recurrent/metastatic tumors owing to newly acquired biological characteristics probably gives tumor cells ability to transmit *via* the circulation or lymphatic systems and metastasize to new sites (38–40). Biomarker discrepancy may contribute to this increased capacity to invade since both endocrine and growth factor signaling pathways are associated with tumor invasion and metastasis (41). Another well-known potential explanation of biomarker discrepancy is selection pressure of treatment (7). There is still short of solid evidence to support this theory. Some studies reported an effect of CT exposure on HoR conversion and of previous anti-HER2 therapy on HER2 conversion (42, 43), while other studies did not find such correlation (12). Here, we found no association between CT and anti-HER2 therapy with ER or HER2 conversion. But we demonstrated a positive correlation between adjuvant ET and PR conversion, which was in favor of this theory. Although we did not observe any association between different ET drugs and PR conversion, further molecular biological studies on these cases will continue to explore the concrete mechanism of the occurrence of biomarker discrepancy.

In terms of the influence of receptor conversion on clinical outcome, Canadian DESTINY study was the biggest prospective study, which enrolled 121 patients with a median follow-up of 12.0 months and they found no significant association between biomarker discrepancy and survival (11). Nevertheless, other two retrospective analyses showed the opposite conclusion. After analyzing data from 789 patients with a median follow-up of 16.8 months, Liedtke et al. identified that cases with biomarker discrepancy between primary and recurrent/metastatic lesions had a significantly worse prognosis (44). Similar conclusion was declared by Dieci et al. in a 119-patient study that patients with biomarker discrepancy had worse PRS and OS (8). The different results among these studies may attribute to different definition of receptor positivity and conversion. Besides, in these studies, different end points were adopted to evaluate clinical outcome. In our study, we did not find significant relationship between biomarker discrepancy and disease outcome. Meanwhile, we demonstrated that primary ER-negative tumor (*P =* 0.014) or distant metastasis site (viscera, *P* < 0.001; bone or soft tissues, *P =* 0.005) were independently associated with worse PRS in multivariate model.

Several studies had evaluated whether biomarker discrepancy would potentially influence subsequent systemic treatment. However, the discordance rates of ER (7-32%), PR (24-54%), and HER2 (1-34%) (24, 45–51) were variable in previous studies according to retrospective data, small populations, heterogeneity of enrolled patients and variabilities of recurrent sites. A pooled analysis (52) of two prospective studies, British BRITS study (12) and the DESTINY study (11), demonstrated that discordant rates of ER, PR, and HER2 status were 12.6%, 31.2%, and 5.5%, respectively. Around one in nine (N=32) patients changed their subsequent systemic treatment based on new receptor status. Unfortunately, impact of biomarker discrepancy on disease outcome was not analyzed in the report. In our study, a similar rate (9.6%) of patients changed following systemic treatment after recurrence. Nevertheless, we did not find any evidence that changing subsequent systemic treatment depending on new receptor statuses had influence on disease outcome after relapse.

To note, our current study included 156 recurrent/metastatic breast cancer patients from 5856 continuous single-center patients, to evaluate biomarker discrepancy between primary and recurrent/metastatic breast cancer lesions and its influence on following treatment and prognosis. However, there are still several limitations. First of all, the retrospective nature of the current study might lead to selection bias and less representativeness of our work. The difficulty of re-biopsy varies among different locations of relapse tumor, which may lead to potential bias. In our daily practice, breast cancer relapse was diagnosed on account of radiological examination or histo-pathological result. Therefore, only patients with “observable” or “evaluable” lesion(s) could be involved, which might cause the bias in time and location of relapse diagnosis. Besides, HER2 status was not available in 52 patients, mainly as a result of no further FISH test following IHC 2+ due to social-economical concerns and restriction of restricted re-biopsy sample quality. These 52 patients were excluded from the analysis of HER2 conversion, which may possibly cause bias. What’s more, although we recommended a re-biopsy of the recurrent lesion for each applicable recurrent/metastatic patient in our actual practice, the real-world proportion of analyzable recurrent/metastatic patients was relatively low due to patient refusal or technological hurdles, which was similar to previous report (47, 53). Last but not least, number of enrolled patients was limited and follow-up period after relapse was relatively short to detect the impact factors for PRS or following systemic treatment, warranting more patients and longer follow-up to draw a more solid conclusion.

In conclusion, biomarker discrepancy was observed between primary and recurrent/metastatic breast cancer lesions and had certain influence on systemic treatment strategies after disease relapse. But its impact on disease outcome was not found. Primary ER-negative and distant metastasis were independently associated with worse PRS in recurrent/metastatic breast cancer patients. Our results provided new insights with regards to the biomarker discrepancy in breast cancer recurrence or metastasis and systemic treatment decision. Further clinical evaluation with a larger cohort and longer follow-up and further translational research are warranted to establish its impact on disease outcomes.

## Data Availability Statement

The original contributions presented in the study are included in the article/**Supplementary Material**. Further inquiries can be directed to the corresponding authors.

## Ethics Statement

This study was reviewed and approved by the independent Ethical Committees of Ruijin Hospital, Shanghai Jiaotong University School of Medicine. All procedures involving human participants were consistent with the ethical standards of the institutional and/or national research committee and with the 1964 Helsinki declaration and its later amendments or comparable ethical standards. Informed consent was obtained from all individual participants included in the study.

## Author Contributions

All authors listed have made a substantial, direct, and intellectual contribution to the work and approved it for publication.

## Funding

The authors received financial supported from the National Natural Science Foundation of China (Grant Number: 81772797), Shanghai Municipal Education Commission—Gaofeng Clinical Medicine Grant Support (20172007), and Ruijin Hospital, Shanghai Jiao Tong University School of Medicine—”Guangci Excellent Youth Training Program” (GCQN-2017-A18). All these financial sponsors had no role in the study design, information collection, data analysis or interpretation.

## Conflict of Interest

The authors declare that the research was conducted in the absence of any commercial or financial relationships that could be construed as a potential conflict of interest.
