# Supplementary figures and images for: Association of Biomarker Discrepancy and Treatment Decision, Disease Outcome in Recurrent/Metastatic Breast Cancer Patients
Source: Front Oncol. 2021 Jul 1;11:638619. doi: 10.3389/fonc.2021.638619 (PMC8283966; doi:10.3389/fonc.2021.638619)

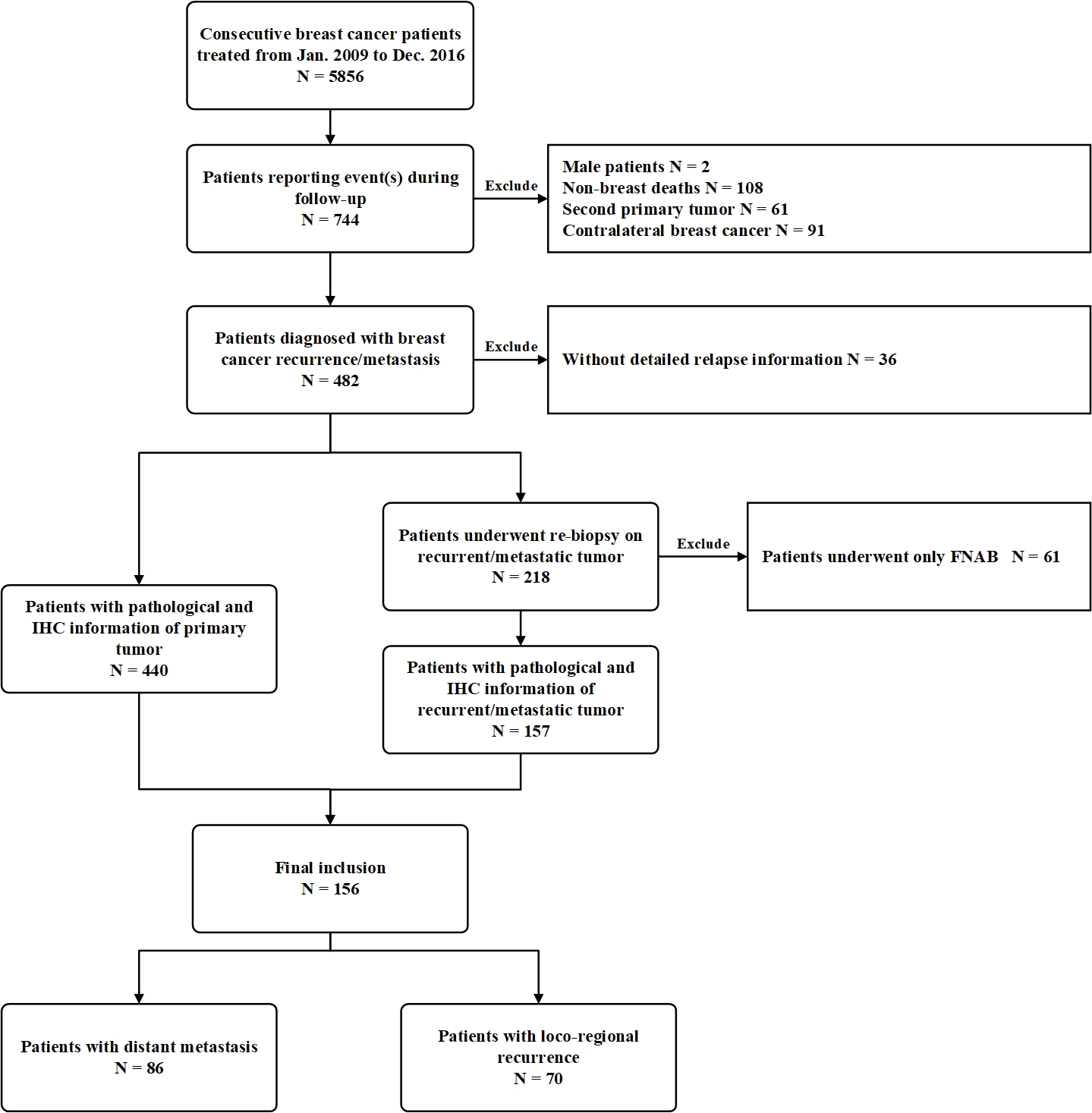

Supplement: Supplementary file 1 [file Image_1.jpeg]
