# Supplementary material for: Association of Biomarker Discrepancy and Treatment Decision, Disease Outcome in Recurrent/Metastatic Breast Cancer Patients
Source: Front Oncol. 2021 Jul 1;11:638619. doi: 10.3389/fonc.2021.638619 (PMC8283966; doi:10.3389/fonc.2021.638619)
Supplement: Supplementary file 3 [file Table_1.docx]

**Supplementary Table S1. Treatment information of patients received neoadjuvant therapy**

| **Neoadjuvant treatment** | **N** | **%** | **ER** | | |  | | **PR** | |  | **HER2** | |
| --- | --- | --- | --- | --- | --- | --- | --- | --- | --- | --- | --- | --- |
|  |  |  | **Gain** | **Loss** |  | | **Gain** | | **Loss** |  | **Gain** | **Loss** |
| **Total** | 30 | 100.0 | 2 | 4 |  | | 4 | | 8 |  | 1 | 0 |
| **CT** | 27 | 90.0 | 1 | 4 |  | | 2 | | 8 |  | 1 | 0 |
| **CT + anti-HER2** | 1 | 3.3 | 1 | 0 |  | | 0 | | 0 |  | NA | NA |
| **CT + ET** | 1 | 3.3 | 0 | 0 |  | | 1 | | 0 |  | 0 | 0 |
| **ET** | 1 | 3.3 | 0 | 0 |  | | 1 | | 0 |  | 0 | 0 |

Abbreviations: ER, estrogen receptor; PR, progesterone receptor; HER2, human epidermal growth factor receptor-2; CT, chemotherapy; ET endocrine therapy; NA, not available

**Supplementary Table S2. Univariate analysis of factors associated with ER, PR and HER2 conversion between primary and recurrent lesions**

| **Characteristics** | **ER** | **PR** | **HER2** |
| --- | --- | --- | --- |
|  | ***P* value** | ***P* value** | ***P* value** |
| **Age** | **0.036** | 0.647 | 0.651 |
| **Menstrual status** | **0.031** | 0.496 | 0.535 |
| **Neoadjuvant treatment** | 0.586 | 0.389 | 0.493 |
| **Breast surgery** | 0.146 | 1.000 | 0.263 |
| **Axillary surgery** | **0.027** | 0.208 | 0.985 |
| **Histological type** | 0.231 | 0.109 | 0.454 |
| **Histological grade** | 0.908 | **0.030** | 0.165 |
| **pT** | 0.520 | 0.195 | 0.724 |
| **pN** | **0.018** | 0.308 | 0.535 |
| **Molecular subtype** | 0.381 | 0.063 | 0.375 |
| **Recurrence site** | 0.347 | 0.782 | 0.401 |
| **DFI** | 0.706 | 0.080 | 0.811 |

Abbreviations: pT, pathological tumor size stage; pN, pathological lymph node stage; ER, estrogen receptor; PR, progesterone receptor; HER2, human epidermal growth factor receptor-2; DFI, disease-free interval

**Supplementary Table S3. Univariate analysis of association between adjuvant therapy and ER, PR, HER2 conversion**

| **Adjuvant therapy** | **ER conversion** | | |  | **PR conversion** | | |  | **HER2 conversion** | | |
| --- | --- | --- | --- | --- | --- | --- | --- | --- | --- | --- | --- |
|  | **Remain** | **Discordant** | ***P* value** |  | **Remain** | **Discordant** | ***P* value** |  | **Remain** | **Discordant** | ***P* value** |
| **Chemotherapy** |  |  | 0.076 |  |  |  | 0.542 |  |  |  | 0.138 |
| Yes | 93 | 14 |  |  | 73 | 34 |  |  | 73 | 3 |  |
| No | 37 | 12 |  |  | 31 | 18 |  |  | 28 | 0 |  |
| **Endocrine therapy** |  |  | 0.113 |  |  |  | **0.012** |  |  |  | 0.124 |
| Yes | 68 | 18 |  |  | 50 | 36 |  |  | 51 | 3 |  |
| No | 62 | 8 |  |  | 54 | 16 |  |  | 50 | 0 |  |
| **Radiotherapy** |  |  | 0.558 |  |  |  | 0.908 |  |  |  | 0.093 |
| Yes | 77 | 17 |  |  | 63 | 31 |  |  | 67 | 1 |  |
| No | 53 | 9 |  |  | 41 | 21 |  |  | 34 | 2 |  |
| **Anti-HER2 therapy** |  |  | 0.173 |  |  |  | 0.100 |  |  |  | 0.219 |
| Yes | 33 | 10 |  |  | 33 | 10 |  |  | 33 | 2 |  |
| No | 97 | 16 |  |  | 71 | 42 |  |  | 68 | 1 |  |

**Supplementary Table S4. Multivariate analysis of factors associated with ER conversion in breast cancer patients**

| **Clinico-pathologic characteristics** | **OR** | **95%CI** | ***P* value** |
| --- | --- | --- | --- |
| **Age** |  |  | 0.664 |
| <50 | 1.00 |  |  |
| ≥50 | 0.73 | 0.18 - 2.95 |  |
| **Menstrual status** |  |  | 0.412 |
| pre/peri | 1.00 |  |  |
| post | 0.56 | 0.14 - 2.25 |  |
| **pN** |  |  | **0.035** |
| 0, x | 1.00 |  |  |
| 1-3 | 0.36 | 0.14 - 0.93 |  |
| **Axillary surgery** |  |  | 0.396 |
| ALND | 1.00 |  |  |
| SLNB | 1.06 | 0.35 - 3.23 | 0.923 |
| None | 3.43 | 0.53 - 22.03 | 0.194 |

Abbreviations: OR, odds ratio; CI, confidence interval; ER, estrogen receptor; pN, pathological lymph node stage; ALND, axillary lymph node dissection; SLNB, sentinel lymph node biopsy; PR, progesterone receptor.

**Supplementary Table S5. Multivariate analysis of factors associated with PR conversion in breast cancer patients**

| **Clinico-pathologic characteristics** | **OR** | **95%CI** | ***P* value** |
| --- | --- | --- | --- |
| **Histological grade** |  |  | 0.199 |
| I-II | 1.00 |  |  |
| III | 0.63 | 0.31 - 1.28 |  |
| **Adjuvant endocrine therapy** |  |  | **0.017** |
| Yes | 1.00 |  |  |
| No | 0.41 | 0.20 - 0.85 |  |

**Supplementary Table S6. Univariate analysis of impact factors on post-recurrence survival**

| **Clinico-pathologic characteristics** | ***P* value** |
| --- | --- |
| **Age** | 0.985 |
| **Menstrual status** | 0.858 |
| **Breast surgery** | **0.005** |
| **Axillary surgery** | **0.013** |
| **Histological grade** | 0.858 |
| **Histological type** | 0.924 |
| **pT** | **0.019** |
| **pN** | 0.153 |
| **ER status** | **0.007** |
| **PR status** | 0.163 |
| **HER2 status** | 0.142 |
| **Ki67 status** | 0.916 |
| **Recurrent site** | **0.014** |
| **ER conversion** | **0.005** |
| **PR conversion** | **0.002** |
| **DFI** | **0.042** |

Abbreviations: pT, pathological tumor size stage; pN, pathological lymph node stage; ER, estrogen receptor; PR, progesterone receptor; HER2, human epidermal growth factor receptor-2; DFI, disease-free interval

**Supplementary Table S7. Subsequent systemic treatment and clinical outcome in the patient with biomarker discrepancy**

|  | **Treatment changed** | |  | **Treatment unchanged** | |
| --- | --- | --- | --- | --- | --- |
|  | **N** | **2-year PRS rate** |  | **N** | **2-year PRS rate** |
| **HoR, N = 25** | 13 | 48.1% |  | 12 | 90.0% |
| Gain | 5 | 75.0% |  | 8 | 100.0% |
| Loss | 8 | 50.0% |  | 4 | 75.0% |
| **HoR+HER2, N = 28** | 15 | 48.1% |  | 13 | 90.0% |
| Gain | 7 | 75.0% |  | 9 | 100.0% |
| Loss | 8 | 50.0% |  | 4 | 75.0% |

Abbreviations: PRS, post-recurrence survival; HoR, hormone receptor; HER2, human epidermal growth factor receptor-2.

.
